# 

**Published:** 1915-05

**Authors:** 


### CONTENTS.

ORIGINAL COMMUNICATIONS—

Technique of X-Ray in Dermatology by Dr. Cosby Swanson, Atlanta, Ga. ........ 57

Three Surgical Myths by Thos. H. Hancock, Atlanta, Ga. 63

The Small Fibrous Prostate by A. L. Fowler, M. D., Atlanta, Ga. ........ 67

A Case of Gun Shot Wound of the Back, Producing Paralysis, Relieved by Laminectomy by T. C. Davidson, M. D., Atlanta, Ga. ........ 71

Meeting of the Fulton County Medical Society by R. R. Daly, M. D. ........ 73

EDITORIAL ........ 89

NEWS AND NOTES ........ 93

SELECTIONS AND ABSTRACTS ........ 97

